# Error in Text

**DOI:** 10.1001/jamahealthforum.2024.3633

**Published:** 2024-10-04

In the Viewpoint titled “Medicare Advantage Supplemental Benefits for Dual-Eligible Beneficiaries and Recommendations for Reform,”^1^ which published September 6, 2024, there was an error in text. In the last sentence in the third paragraph of the Opportunities for Reform section, the fifth reference should have been cited. This article has been corrected.^1^
